# A novel Bayesian Max-EWMA control chart for jointly monitoring the process mean and variance: an application to hard bake process

**DOI:** 10.1038/s41598-023-48532-4

**Published:** 2023-12-01

**Authors:** Javed Iqbal, Muhammad Noor-ul-Amin, Imad Khan, Salman A. AlQahtani, Uzma Yasmeen, Bakhtyar Ahmad

**Affiliations:** 1 COMSATS University Islamabad, Lahore Campus, Islamabad, Pakistan; 2 Abdul Wali Khan University Mardan, Mardan, Pakistan; 3 Computer Engineering Department, College of Computer and Information Sciences, King Saud University, Riyadh, Saudi Arabia; 4 Department of Mathematics & Statistics, BROCK University, St. Catharines, Canada; 5 Higher Education Department Afghanistan, Kabul, Afghanistan

**Keywords:** Mechanical engineering, Scientific data, Statistics

## Abstract

In this article, we introduce a novel Bayesian Max-EWMA control chart under various loss functions to concurrently monitor the mean and variance of a normally distributed process. The Bayesian Max-EWMA control chart exhibit strong overall performance in detecting shifts in both mean and dispersion across various magnitudes. To evaluate the performance of the proposed control chart, we employ Monte Carlo simulation methods to compute their run length characteristics. We conduct an extensive comparative analysis, contrasting the run length performance of our proposed charts with that of existing ones. Our findings highlight the heightened sensitivity of Bayesian Max-EWMA control chart to shifts of diverse magnitudes. Finally, to illustrate the efficacy of our Bayesian Max-EWMA control chart using various loss functions, we present a practical case study involving the hard-bake process in semiconductor manufacturing. Our results underscore the superior performance of the Bayesian Max-EWMA control chart in detecting out-of-control signals.

## Introduction

Embedded at the core of industries committed to unparalleled process excellence and quality assurance, Statistical Process Control (SPC) serves as a foundational strategy for achieving process excellence. It is built on meticulous statistical analysis, enabling robust monitoring, analysis, and optimization in alignment with benchmarks. By leveraging data insights, SPC adeptly discerns process variations, facilitating informed decisions and swift interventions. Framed within statistical methodologies, it deftly navigates process intricacies, promoting efficiency, defect reduction, and exceeding customer expectations. Applicable across sectors, SPC principles guide the intricate path of process refinement and continuous enhancement. At its core, the control chart (CC) is a key SPC component, facilitating ongoing monitoring and insightful process data analysis. Plotting data against control limits reveals trends and anomalies, enabling agile interventions for stability and quality assurance. The graphical representation offered by control charts serves as a guiding light in the realm of decision-making, all while nurturing a culture deeply rooted in perpetual process enhancement. Shewhart^1^ introduced CCs that exclusively employ current sample data to identify substantial variations within production processes. In contrast, memory-type CCs such as cumulative sum (CUSUM) and exponentially weighted moving average (EWMA) CCs were pioneered by Page^2^ and Roberts^3^, encompassing both current and historical sample data. It's noteworthy that CUSUM and EWMA CCs demonstrate heightened sensitivity in detecting slight to moderate shifts in process parameters compared to the traditional Shewhart CCs. These memory-type CCs, particularly CUSUM and EWMA, find extensive utilization across diverse domains, prominently in chemical and industrial production processes. Herdiani et al.^4^ noted that in SPC, the assumption of independent observations frequently becomes invalid, necessitating specialized CCs like Shewhart, CUSUM, and EWMA for correlated data. The inclusion of time series models becomes pivotal due to this correlation. Shewhart's modification for autoregressive processes incorporated the mean-to-target distance relative to the standard deviation of the autocorrelation process. The study assesses the EWMA mean for autocorrelation processes, with a focus on evaluating performance through the ARL utilizing the Markov Chain Method. Gan^5^ evaluated control-charting schemes for joint monitoring of process mean and variance, exposing their limitations and the risk of individual application. A combined two-sided EWMA CC jointly monitoring of mean and variance demonstrated efficacy across various out-of-control scenarios, with the study presenting a method for approximating average run length (ARL) and run-length distribution percentage points, along with a suggested design procedure for the combined EWMA scheme. Sanusi et al.^6^ compare four EWMA scheme combinations for jointly identifying Gaussian process mean and variance, addressing parameter estimation via maximum likelihood estimators. Distance-type schemes outperform certain existing methods in identifying slight-to-moderate shifts, supported by computational studies and real industrial data. Haq and Razzaq^7^ examined MaxWACCUSUM CC using three unbiased estimators for joint monitoring of mean and variance shifts, showing strong performance in detecting various shifts and outperforming existing charts like maximum EWMA and CUSUM, supported by Monte Carlo simulations and real datasets. Arif et al.^8^ assessed the effects of measurement errors (ME) on a joint monitoring CC using three techniques. They explored the chart's application with EWMA statistics and generalized likelihood ratio tests in ranked set sampling. The study evaluated the chart's performance through simulations involving various shifts, and it was further supported by a real-world data example. Javiad et al.^9^ investigated the effects of ME on monitoring mean and variance shifts in production processes. They analyzed Max-EMWA CC, employing a covariate model and multiple measurements to counter ME effects. Monte Carlo simulations were utilized to compute ARLs and SDRLs, and a real-world data example was used to validate findings and compare with other chart methods. Yang^10^ introduced an enhanced quality optimization approach, the Qpm MQCAC, which monitors shifts in process mean and standard deviation, ensuring quality adherence, efficient resource usage, and alignment with green manufacturing objectives. Noor-ul-Amin et al.^11^ introduced a Max-EWMA CC utilizing the inverse response function for simultaneous monitoring of process mean and dispersion in Weibull-distributed processes, demonstrating increased sensitivity compared to existing Max-EWMA CC, validated through ARL and SDRL metrics, and supported by practical examples. Earlier studies suggest a widespread reliance on conventional techniques that exclusively utilize sample data, often overlooking prior information. On the contrary, the Bayesian method combines sample data with previous knowledge to revise and establish a posterior distribution (P), thereby improving the estimation procedure. Noor-ul-Amin and Noor^12^ developed a new AEWMA CC for Bayesian process mean monitoring, investigating it under different LFs and informative priors. They conducted a comparative study with existing Bayesian EWMA CCs, utilizing run length as performance metrics, and substantiated their discoveries using Mnote Carlo simulation alongside a real-world data illustrations. Raiz et al.^13^ studied the Bayesian EWMA CC under three LFs (SELF, LLF, PLF) and diverse informative and non-informative priors. Performance metrics like ARL and SDRL, computed via P distributions, assess the chart's effectiveness. Monte Carlo simulations explore performance across smoothing constants, alongside an illustrative example highlighting practical use cases. Bayesian EWMA CCs for non-normal lifetime distributions, i.e., Exponential, Inverse Rayleigh, and Weibull distributions, are suggested by Noor et al.^14^. They utilized uniform priors with LFs, evaluating charts using ARL and SDRL. Through simulations, the Weibull-based chart demonstrated superior performance, corroborated by a real data illustration. Noor et al.^15^ used Bayesian methods to develop a hybrid EWMA CC, considering informative and non-informative priors along with two LFs. They evaluated performance using ARL and SDRL through posterior and predictive posterior distributions. Extensive simulations and a real-data example validated their approach. Ranked set sampling (RSS) is a statistical approach that improves estimating population parameters by ranking items within a population based on specific characteristics and selecting sets or groups of items according to their ranks, rather than through individual random selections. This method helps in scenarios with high measurement costs or significant population variation by reducing estimation variability through the use of entire subsets of items. Utilizing ranked sets enhances the precision of population parameter estimations, potentially providing cost-effective sampling strategies in diverse research or data collection contexts. Khan et al.^16^ introduce a Bayesian hybrid EWMA CC via RSS with informative priors and different loss functions (LFs). Their simulation-based evaluation using ARL and SDRL highlights its superiority in identifying out-of-control signals in semiconductor manufacturing compared to other Bayesian CCs. Aslam and Anwar^17^ developed a Bayesian Modified-EWMA CC for process location monitoring, integrating four LFs and a conjugate prior. The chart's effectiveness in detecting small to moderate shifts is demonstrated through performance assessment and real-life instances, including monitoring mechanical reaming and sports industry golf ball performance. Khan et al.^18^ proposed a novel Bayesian AEWMA CC using RSS and informative prior for mean shift monitoring. Extensive Monte Carlo simulations revealed its improved sensitivity in detecting mean shifts compared to existing Bayesian AEWMA charts based on SRS. Illustrated with a semiconductor fabrication example, it outperformed EWMA and AEWMA CCs using Bayesian approach under SRS for detecting out-of-control signals.

In manufacturing, Bayesian statistics plays a pivotal role, leveraging prior knowledge for informed parameter inferences and maintaining process control by dynamically updating parameters amidst changing conditions. This adaptability is crucial given the intricate nature of manufacturing processes. Bayesian methods excel in handling uncertainty, representing it through probability distributions for effective risk management and decision-making. Their strength lies in continuous monitoring via sequential analysis, enabling early fault detection and corrective actions. Their flexibility accommodates various manufacturing scenarios, integrating seamlessly into control systems for optimization, waste reduction, and quality maintenance. Offering a clear decision-making framework amid uncertainty, Bayesian modeling aids root cause analysis, contributing significantly to improved product quality, efficiency, and overall process control. Additionally, this article introduces a novel Bayesian Max-EWMA CC for simultaneous monitoring of both process mean and variance. The method's performance evaluation involves ARL and SDRL calculations, executed via Monte Carlo simulation techniques. The structure of the article is as follows: In “Bayesian approach” section, we introduce Bayesian theory and various LFs. In “Proposed Bayesian Max-EWMA CC for joint monitoring” section, we discuss the proposed Bayesian Max-EWMA CC method. Following that, "Performance evaluation" section comprises comprehensive discussions and emphasizes key findings, while “Real life data application” section provides practical illustrations of real-life data applications. Finally, “Conclusion” section serves as the conclusion of this article.

## Bayesian approach

Bayesian theory, a foundational concept in statistics and probability, provides a unique and powerful framework for making inferences and drawing conclusions from data. Unlike traditional frequentist statistics, where parameters are considered fixed and unknown, Bayesian theory treats these parameters as probability distributions, allowing us to incorporate prior knowledge and update our beliefs as new evidence emerges. These prior distributions can be broadly categorized into two groups: non-informative and informative. Non-informative priors, such as Jeffreys and uniform priors, are commonly used, while informative priors frequently rely on conjugate priors, which are a widely recognized family of distributions. This approach not only offers a flexible and intuitive way to analyze data but also provides valuable insights into uncertainty, making it a fundamental tool in various fields, including science, engineering, and machine learning. Let's examine the study variable *X* within the  confines of a controlled process, delineated by parameters $\theta$ (mean) and $\delta^{2}$ (variance). In this scenario, we employ a normal prior, with $\theta_{0}$ and $\delta_{0}^{2}$ serving as its associated parameters, defined as follows:

1 $$p\left( \theta \right) = \frac{1}{{\sqrt {2\pi \delta_{0}^{2} } }}\exp \left\{ { - \frac{1}{{2\delta_{0}^{2} }}\left( {\theta - \theta_{0} } \right)^{2} } \right\}$$

Generate the P distribution, it involves combining the likelihood function from the sample distribution with the prior distribution, forming a proportional relationship via multiplication. Consequently, the resulting P distribution, delineating the unknown parameter $\theta$ based on the observed data *X*, can be expressed as follows:

2 $$p\left( {\theta |x} \right) = \frac{{p\left( {x|\theta } \right)p\left( \theta \right)}}{{\int {p\left( {x|\theta } \right)p\left( \theta \right)d\theta } }}$$

The posterior predictive (PP) distribution is employed to predict future observations by considering the P distribution as prior distribution. It is frequently employed as a prior distribution for new data *Y*, facilitating predictions for upcoming observations while taking uncertainty into account. An integral component of Bayesian theory, the PP distribution enables the updating of prior distributions with new data. Its mathematical illustration is given below:

3 $$p\left( {y|x} \right) = \int {p\left( {y|\theta } \right)p\left( {\theta |x} \right)d\theta }$$

### Squared error loss functions

In Bayesian estimation, the squared error LF (SELF) is a crucial tool for assessing the accuracy of parameter estimates. It measures the discrepancy between estimated and true values by squaring the difference between them. Bayesian estimation combines prior beliefs and observed data to infer unknown parameters. The SELF penalizes larger estimation errors more severely than smaller ones. The goal is to find the Bayesian posterior mean, minimizing the expected squared error under the posterior distribution. This approach leads to robust estimates, particularly when the posterior is approximately Gaussian. In this study, we employed Gauss's recommended LF^19^. The SELF, which considers both the variable of interest, denoted as *X*, and the estimator $\hat{\theta }$ for the unknown population parameter $\theta$, is expressed mathematically as follows:

4 $$L\left( {\theta ,\hat{\theta }} \right) = \left( {\theta - \hat{\theta }} \right)^{2}$$

and Bayes estimator utilizing SELF is mathematized as:

5 $$\hat{\theta }_{{\left( {SELF} \right)}} = E_{\theta /x} \left( \theta \right).$$

### Linex loss functions

An asymmetric LF in Bayesian analysis quantifies the penalties for incorrect predictions, unlike a symmetric one that treats errors equally. It assigns different weights to overestimations and underestimations based on their relative costs, incorporating prior beliefs about data distribution and outcomes' costs to improve Bayesian inference precision and efficiency. Varian^20^ proposed the LLF to mitigate risks in Bayes estimation. The LLF is mathematically described as:

6 $$L\left( {\theta ,\hat{\theta }} \right) = \left( {e^{{c\left( {\theta - \hat{\theta }} \right)}} - c\left( {\theta - \hat{\theta }} \right) - 1} \right)$$

Under LLF, the Bayesian estimator $\hat{\theta }$ is mathematizied as

7 $$\hat{\theta }_{{\left( {LLF} \right)}} = - \frac{1}{c}InE_{\theta /x} \left( {e^{ - c\theta } } \right).$$

## Proposed Bayesian Max-EWMA CC for joint monitoring

In this section, we introduce the Max-EWMA Control Chart, which leverages Bayesian theory to concurrently monitor the mean and variance of a normally distributed process. Let *X*_1_, *X*_2_, … *X*n represent independent and identically normally distributed random variables with a mean of *θ* and a variance of $\sigma^{2}$. The corresponding probability function is mathematically expressed as:

8 $$f\left( {x_{t} :\theta ,\sigma^{2} } \right) = \frac{1}{{\sqrt {2\pi \sigma^{2} } }}\exp \left( { - \tfrac{1}{{2\sigma^{2} }}\left( {x_{t} - \theta } \right)^{2} } \right).$$

In a Bayesian framework, if the likelihood function and prior distribution are both normally distributed, the resulting posterior distribution also conforms to a normal distribution, with a mean (θ) and variance (σ). The pdf is as follows:

9 $$P\left( {\theta /x} \right) = \frac{1}{{\sqrt {2\pi } \sqrt {\frac{{\sigma^{2} \sigma_{0}^{2} }}{{\sigma^{2} + n\sigma_{0}^{2} }}} }}\exp \left[ { - \frac{1}{2}\left( {\frac{{\theta - \sum\limits_{i = 1}^{n} {\frac{{x_{i} \sigma_{0}^{2} + \theta_{0} \sigma_{0}^{2} }}{{\sigma^{2} + n\sigma_{0}^{2} }}} }}{{\sqrt {\frac{{\sigma^{2} \sigma_{0}^{2} }}{{\sigma^{2} + n\sigma_{0}^{2} }}} }}} \right)^{2} } \right]$$

where $\theta_{n} = \frac{{n\overline{x} \sigma_{0}^{2} + \sigma^{2} \theta_{0} }}{{\sigma^{2} + n\sigma_{0}^{2} }}$ and $\sigma_{n}^{2} = \frac{{\sigma^{2} \sigma_{0}^{2} }}{{\sigma^{2} + n\sigma_{0}^{2} }}$ respectively.

To create a Max-EWMA chart using Bayesian methodology, we begin by selecting a sample of *n* values for a quality characteristic *X* from the production process. Subsequently, we compute transformed statistics under SELF for both the mean and variance as follows

10 $$U_{t} = \frac{{\hat{\theta }_{(SELF)} - \theta }}{{{\raise0.7ex\hbox{$\delta $} \!\mathord{\left/ {\vphantom {\delta {\sqrt n }}}\right.\kern-0pt} \!\lower0.7ex\hbox{${\sqrt n }$}}}}$$

and

11 $$V_{t} = \phi^{ - 1} \left[ {H\left\{ {\frac{{\left( {n - 1} \right)\hat{\delta }_{(SELF)}^{2} }}{{\delta^{2} }}} \right\},\left( {n - 1} \right)} \right]$$

where $\hat{\theta }_{(SELF)} = \frac{{n\overline{x} \sigma_{0}^{2} + \sigma^{2} \theta_{0} }}{{\sigma^{2} + n\sigma_{0}^{2} }}$ and $\hat{\sigma }_{(SELF)}^{2} = \frac{{\sigma^{2} \sigma_{0}^{2} }}{{\sigma^{2} + n\sigma_{0}^{2} }}$ are the Bayes estimators using SELF for the population mean and variance respectively, while using LLF the Bayesian estimators for population mean and variance is given as $\hat{\theta }_{{\left( {_{LLF} } \right)}} = \frac{{n\overline{x}_{{(RSS_{i} )}} \sigma_{0}^{2} + \sigma^{2} \theta_{0} }}{{\sigma^{2} + n\sigma_{0}^{2} }} - \frac{{C^{\prime} }}{2}\sigma_{n}^{2}$ and $\hat{\sigma }_{(LLF)}^{2} = \frac{{\sigma^{2} \sigma_{0}^{2} }}{{\sigma^{2} + n\sigma_{0}^{2} }}$, the transform statistic under LLF for both the process mean and variance is mathematically described as:

12 $$U_{t} = \frac{{\hat{\theta }_{(LLF)} - \theta }}{{{\raise0.7ex\hbox{$\sigma $} \!\mathord{\left/ {\vphantom {\sigma {\sqrt n }}}\right.\kern-0pt} \!\lower0.7ex\hbox{${\sqrt n }$}}}}$$

and

13 $$V_{t} = \phi^{ - 1} \left[ {H\left\{ {\frac{{\left( {n - 1} \right)\hat{\sigma }_{(LLF)}^{2} }}{{\sigma^{2} }}} \right\},\left( {n - 1} \right)} \right]$$

where $H\left( {n,\nu } \right)$ is a chi-square distribution characterized by $\nu$ degrees of freedom, and $\phi^{ - 1}$ denotes the inverse of the standard normal distribution function, the computations for EWMA statistics regarding both the process mean and variance are outlined as follows:

14 $$P_{t(LF)} = \lambda U_{t(LF)} + \left( {1 - \lambda } \right)P_{t - 1(LF)}$$

15 $$Q_{t(LF)} = \lambda V_{t(LF)} + \left( {1 - \lambda } \right)V_{t - 1(LF)}$$

In this context,$P_{0}$ and $Q_{0}$ represent the initial values for the EWMA sequences *P*_*t*_ and *Q*_*t*_, respectively, with $\lambda$ (a constant within the range [0, 1]) denoting the smoothing constant. *P*_*t*_ and *Q*_*t*_ are also mutually independent due to the independence of *P*_*t*_ and *Q*_*t*_. When considering an in-control process, both *P*_*t*_ and *Q*_*t*_ follow normal distributions, each with a mean of zero and variances of $\delta_{{P_{t} }}^{2}$ and $\delta_{{Q_{t} }}^{2}$, respectively. This is defined as follows

16 $$\delta_{{P_{t} }}^{2} = \delta_{{Q_{t} }}^{2} = \frac{\lambda }{\lambda - 1}\left[ {1 - \left( {1 - \lambda } \right)^{2t} } \right],\quad {\text{for}}\quad t = 1,2, \ldots$$

The plotting statistics, Bayesian Max-EWMA for jointly monitoring using $P_{t(LF)}$ and $Q_{t(LF)}$ is mathematically defined as:

17 $$Z_{t} = Max\left( {\left| {P_{t(LF)} } \right|,\left| {Q_{t(LF)} } \right|} \right),$$

For $$t = 1,2, \ldots$$

As the Bayesian Max-EWMA statistic is positive value, so we required to plot only the upper control limit for jointly monitoring of the process mean and variance, if the plotting statistic $Z_{t}$ within the UCL, then the process is in-control and if the $Z_{t}$ cross the UCL the process is out-of-control.

## Performance evaluation

The performance of the proposed control charts has been assessed using Average Run Length (ARL) and Standard Deviation of Run Length (SDRL) as the key metrics. These metrics serve as the benchmark for evaluating the effectiveness of the control charts. The baseline ARL (*ARL*_0_) and SDRL (*SDRL*_0_) values represent the performance under normal, in-control conditions. On the other hand, *ARL*_1_ and *SDRL*_1_ denote the ARL and SDRL values, respectively, when the process deviates from the normal, indicating an out-of-control situation. The evaluation encompasses various mean shifts to provide a comprehensive understanding of the proposed charts' performance characteristics in different scenarios. We employed 50,000 replicates to calculate both the ARL and SDRL. The smoothing constants were set at λ = 0.10 and 0.25. Additionally, we explored various combinations of mean shift values, denoted as a = 0.00, 0.25, 0.50, 0.75, 1.00, 1.25, 1.50, 1.75, 2.00, 2.25, 2.50, 2.75, 3.00, as well as variance shift values, denoted as b = 0.25, 0.50, 0.75, 0.90, 1, 1.10, 1.25, 1.50, 2.00, 2.50, 3.00. These combinations were used in our study to assess the performance of the Bayesian Max-EWMA CC method in simultaneously monitoring both process mean and variance. The following simulation steps have been considered for the calculations of ARLs and SDRLs.

*Step 1* Establishing the control limits

1. Start by setting up the initial control limits, determining the values for UCL and λ.
2. Create a random sample of size *n*, representing the in-control process by using normal distributions.
3. Compute the statistic for the proposed control chart.
4. Check if the plotting statistic falls within the UCL If it does then proceed to steps (iii–iv) again.

*Step 2* Assessing the out-of-control Average Run Length (ARL).

1. Create a random sample for the process with a shift.
2. Compute the statistic of the proposed control chart.
3. If the plotting statistic falls within the UCL, repeat steps (i–ii). Otherwise, record the number of generated points, representing a single out-of-control run length.
4. Repeat the above process (i–iii) 50,000 times to determine the out-of-control ARL_1_ and SDRL_1_.

Tables 1, 2, 3 and 4 provide a framework for showcasing the outcomes derived from implementing the Bayesian Max-EWMA CC technique. This analysis meticulously evaluates the influence of distinct LFs tailored to emphasize the P and distributions, all assessed within the framework of informative priors. Based on the findings, the suggested Bayesian Max-EWMA CC designed for the simultaneous monitoring of production processes demonstrates an elevated degree of sensitivity when it comes to identifying signs of being out of control. Tables 1 and 2 provide compelling evidence that the Bayesian Max-EWMA CC, particularly under the SELF for P and PP distributions, efficiently detects shifts in both the process mean and variance in tandem. It is noteworthy that with each increment in the magnitude of the mean shift, there is a corresponding reduction in the values of ARLs. Similarly, each variance shift leads to a decrease in ARLs. These observations strongly suggest that this CC possesses the capability to promptly identify process shifts, making it a valuable tool for the early and comprehensive monitoring of production processes. For example, consider the ARL outcomes of the suggested Bayesian Max-EWMA CC when applying the SELF with a smoothing parameter $\lambda = 0.10$, *n* = *5*. i.e., a = 0.00, 0.25, 0.50, 0.75, 1.50, 3.00, while considered the corresponding shift in the process variance i.e., *b* = 1. The resulting ARL values for these shifts were 369.41, 28.27, 9.42, 5.59, 2.67, and 1.51, respectively. It is evident that as the magnitude of the mean shift increase, the ARL values significantly decrease. This observation underscores the higher efficiency of the suggested Bayesian Max-EWMA CC in detecting shifts in the process mean. Similarly, when we examine the impact of varying the process variance *i.e., b* = 0.25, 0.50, 0.75,1, 1.50, 3.00, with process mean *a* = 0.00. The ARL values are 3.39, 6.28, 20.59, 370.34, 7.87, and 1.76., the corresponding ARL values were 3.39, 6.28, 20.59, 370.34, 7.87, and 1.76. These results indicate that when the process variance changes from 1, the ARL outcomes decrease, demonstrating the significant performance of the proposed CC in detecting changes in process variance. Additionally, we observed from Table 2 that the performance of the suggested Bayesian Max-EWMA CC decreases as the smoothing constant values increase. This suggests that a lower smoothing constant may be more effective in certain scenarios.

**Table 1:** ARL and SDRL outcomes for Bayesian Max EWMA CC applying P distribution applying SELF, with $\lambda$ = 0.10.

$\beta$	$n$	$\alpha$ = 0.00		$\alpha$ = 0.10		$\alpha$ = 0.25		$\alpha$ = 0.50		$\alpha$ = 0.75		$\alpha$ = 1.00		$\alpha$ = 1.50		$\alpha$ = 2.00		$\alpha$ = 2.50		$\alpha$ = 3.00	
		ARL	SDRL	ARL	SDRL	ARL	SDRL	ARL	SDRL	ARL	SDRL	ARL	SDRL	ARL	SDRL	ARL	SDRL	ARL	SDRL	ARL	SDRL
0.25	3	5.17	0.91	5.17	0.90	5.15	0.90	5.16	0.92	5.16	0.89	4.82	0.58	3.23	0.42	2.70	0.45	2.00	0.00	2.00	0.00
	5	3.39	0.50	3.38	0.50	3.39	0.51	3.38	0.51	3.38	0.51	3.37	0.49	2.88	0.32	2.00	0.00	2.00	0.00	1.61	0.48
	7	2.82	0.38	2.83	0.38	2.83	0.38	2.83	0.38	2.83	0.38	2.82	0.37	2.00	0.08	2.00	0.00	1.73	0.44	1.72	0.44
0.50	3	10.89	3.83	10.81	3.76	10.83	3.71	10.04	2.83	7.26	1.41	5.24	0.86	3.37	0.50	2.61	0.48	2.01	0.12	1.99	0.01
	5	6.28	1.52	6.26	1.51	6.25	1.51	6.15	1.36	5.14	0.83	3.94	0.57	2.73	0.44	2.00	0.07	1.99	0.09	1.55	0.49
	7	4.80	0.99	4.81	1.00	4.81	1.01	4.76	0.94	4.20	0.62	3.27	0.46	2.12	0.33	1.99	0.03	1.61	0.48	1.61	0.48
0.75	3	47.33	35.21	46.17	33.89	34.63	22.43	13.58	5.51	7.69	2.27	5.32	1.27	3.41	0.63	2.57	0.51	2.07	0.26	1.98	0.13
	5	20.59	11.37	20.57	11.39	17.60	8.36	9.12	2.87	5.57	1.35	3.99	0.81	2.69	0.51	2.04	0.20	1.94	0.22	1.52	0.49
	7	13.79	6.35	13.83	6.26	12.45	4.90	7.21	1.96	4.55	1.01	3.35	0.62	2.22	0.41	1.97	0.15	1.58	0.49	1.58	0.49
0.9	3	247.53	238.18	174.21	161.24	48.54	36.45	13.95	6.64	5.41	1.54	5.43	1.66	3.45	0.76	2.58	0.55	2.11	0.32	1.96	0.20
	5	131.58	120.68	94.68	82.03	28.27	17.97	9.50	3.70	5.61	1.65	4.05	0.97	2.68	0.57	2.07	0.27	1.91	0.28	1.52	0.49
	7	83.45	71.09	62.85	50.21	20.80	11.73	7.50	2.58	4.61	1.19	3.38	0.75	2.26	0.44	1.95	0.22	1.57	0.49	1.56	0.49
1.00	3	369.88	363.60	182.46	172.22	44.89	35.10	13.69	7.05	7.71	2.68	5.43	2.02	3.47	0.84	2.59	0.58	2.13	0.36	1.95	0.25
	5	370.34	367.87	135.66	125.39	28.63	19.93	9.48	3.99	5.60	1.76	4.05	1.06	2.69	0.60	2.09	0.32	1.88	0.32	1.52	0.49
	7	369.77	367.77	104.42	93.23	21.26	13.19	7.51	2.85	4.60	1.30	3.40	0.81	2.27	0.46	1.93	0.27	1.55	0.49	1.55	0.49
1.25	3	33.36	25.67	30.79	23.45	22.18	14.98	13.72	6.97	5.41	2.01	5.42	2.01	3.51	1.03	2.63	0.67	2.17	0.45	1.92	0.36
	5	19.74	12.76	18.54	11.73	14.54	8.30	8.51	3.85	5.58	2.08	4.08	1.29	2.71	0.70	2.12	0.42	1.84	0.39	1.51	0.50
	7	14.52	8.32	13.93	7.83	11.39	5.76	6.93	2.79	4.59	1.51	3.42	1.00	2.33	0.54	1.90	0.37	1.54	0.49	1.55	0.49
1.50	3	12.57	7.54	12.35	7.20	11.29	6.47	12.00	6.48	6.68	3.04	5.21	2.09	3.50	1.20	2.67	0.78	2.19	0.54	1.92	0.44
	5	7.87	3.76	7.80	3.70	7.44	3.40	6.26	2.61	4.92	1.87	3.95	1.38	2.74	0.80	2.14	0.51	1.82	0.45	1.51	0.50
	7	6.10	2.54	6.03	2.49	5.82	2.37	5.10	1.90	4.15	1.40	3.32	1.04	2.34	0.61	1.88	0.45	1.54	0.50	1.54	0.50
2.00	3	5.69	2.75	5.65	2.70	5.53	2.61	8.90	4.53	4.72	2.06	4.19	1.78	3.30	1.26	2.65	0.91	2.20	0.70	1.90	0.59
	5	3.83	1.47	3.81	1.49	3.76	1.42	3.61	1.31	3.43	1.24	3.58	1.25	2.55	0.82	2.11	0.64	1.79	0.55	1.52	0.51
	7	3.07	1.06	3.06	1.03	3.04	1.03	2.97	0.97	2.82	0.91	2.61	0.81	2.20	0.64	1.83	0.54	1.52	0.51	1.53	0.51
2.50	3	3.82	1.71	3.79	1.69	3.75	1.67	3.65	1.57	3.53	1.53	3.32	1.41	2.88	1.17	2.48	0.95	2.15	0.78	1.87	0.67
	5	2.65	0.96	2.67	0.95	2.65	0.92	2.61	0.93	2.54	0.89	2.44	0.86	2.21	0.74	1.94	0.65	1.71	0.58	1.50	0.53
	7	2.20	0.72	2.19	0.70	2.18	0.70	2.18	0.69	2.12	0.67	2.06	0.66	1.89	0.59	1.70	0.55	1.47	0.52	1.46	0.52
3.00	3	2.91	1.26	2.90	1.26	2.92	1.27	2.85	1.22	2.80	1.20	2.72	1.16	2.45	1.02	2.25	0.90	2.01	0.80	1.81	0.70
	5	2.09	0.76	2.12	0.77	2.11	0.76	2.09	0.76	2.06	0.74	2.00	0.71	1.88	0.67	1.75	0.63	1.59	0.58	1.45	0.53
	7	1.76	0.61	1.76	0.60	1.77	0.60	1.74	0.59	1.71	0.59	1.68	0.59	1.60	0.56	1.48	0.53	1.35	0.49	1.36	0.49

**Table 2:** Runlength results for suggested CCBayesian Max EWMA CC applying P distribution under SELF, with $\lambda$ = 0.25.

$\beta$	$n$	$\alpha$ = 0.00		$\alpha$ = 0.10		$\alpha$ = 0.25		$\alpha$ = 0.50		$\alpha$ = 0.75		$\alpha$ = 1.00		$\alpha$ = 1.50		$\alpha$ = 2.00		$\alpha$ = 2.50		$\alpha$ = 3.00	
		ARL	SDRL	ARL	SDRL	ARL	SDRL	ARL	SDRL	ARL	SDRL	ARL	SDRL	ARL	SDRL	ARL	SDRL	ARL	SDRL	ARL	SDRL
0.25	3	4.48	1.19	4.50	1.20	4.50	1.21	4.49	1.20	4.47	1.15	4.01	0.70	2.69	0.45	1.99	0.01	1.93	0.24	1.02	0.15
	5	2.65	0.53	2.66	0.53	2.67	0.53	2.65	0.53	2.64	0.53	2.62	0.49	1.99	0.02	1.84	0.35	1.00	0.01	1.00	0.00
	7	2.05	0.24	2.06	0.26	2.06	0.25	2.06	0.25	7.02	3.12	2.06	0.26	1.99	0.06	1.01	0.10	1.00	0.00	1.00	0.00
0.50	3	14.73	9.49	14.44	9.31	14.21	9.68	13.36	7.90	7.52	2.75	4.55	1.14	2.62	0.53	2.01	0.14	1.77	0.41	1.16	0.36
	5	5.90	2.30	5.93	2.28	5.91	2.27	5.69	2.00	4.38	1.08	3.14	0.60	2.03	0.20	1.69	0.46	1.04	0.20	1.00	0.00
	7	4.08	1.21	4.07	1.23	4.08	1.22	3.98	1.10	4.00	1.10	3.34	0.69	1.93	0.25	1.13	0.33	1.00	0.01	1.00	0.00
0.75	3	115.10	108.75	114.39	108.55	89.77	84.17	21.29	15.58	7.79	3.69	4.63	1.63	2.66	0.67	2.02	0.35	1.70	0.45	1.25	0.43
	5	36.54	30.42	37.17	31.67	31.11	24.95	10.28	5.58	4.85	1.76	3.23	0.91	2.08	0.38	1.63	0.48	1.12	0.32	1.00	0.00
	7	19.69	14.36	19.48	14.35	16.97	11.83	7.04	3.10	4.01	3.11	3.76	1.15	1.84	0.38	1.22	0.41	1.00	0.07	1.00	0.00
0.9	3	275.23	270.77	236.46	227.03	103.1	96.74	18.40	13.69	7.60	4.03	4.65	1.88	2.70	0.78	2.03	0.51	1.67	0.48	1.28	0.45
	5	263.66	259.12	194.64	191.14	53.48	48.85	10.35	6.35	4.92	2.08	3.28	1.07	2.09	0.47	1.61	0.49	1.16	0.37	1.01	0.11
	7	170.00	165.04	130.39	126.10	34.16	28.66	7.29	3.79	7.33	3.82	3.83	1.39	1.82	0.43	1.25	0.43	1.01	0.13	1.00	0.00
1.00	3	369.43	364.18	250.14	241.76	74.13	70.17	16.32	12.05	7.46	4.12	4.68	2.06	2.75	0.86	2.04	0.45	1.65	0.49	1.31	0.46
	5	370.61	369.43	155.66	143.39	44.74	39.57	9.85	6.16	4.96	2.23	3.28	1.16	2.10	0.53	1.60	0.50	1.19	0.39	1.02	0.15
	7	369.87	361.22	161.37	156.70	29.94	25.42	7.20	3.87	7.26	4.04	3.84	1.47	1.80	0.48	1.28	0.45	1.02	0.16	1.00	0.02
1.25	3	36.11	32.76	33.94	30.34	24.08	20.79	11.77	8.35	6.75	3.96	4.56	2.25	2.76	1.02	2.05	0.65	1.65	0.53	1.35	0.48
	5	21.57	18.24	20.41	16.93	14.96	11.42	7.76	4.77	4.70	2.34	3.31	1.37	2.12	0.68	1.60	0.53	1.24	0.43	1.06	0.23
	7	15.16	11.62	14.35	10.83	11.14	7.96	6.03	3.36	6.03	3.33	3.75	1.65	1.80	0.56	1.33	0.47	1.06	0.23	1.00	0.05
1.50	3	11.46	8.61	11.17	8.41	10.22	7.49	7.70	5.11	5.57	3.31	4.18	2.19	2.76	1.16	2.06	0.75	1.65	0.59	1.38	0.50
	5	6.86	4.37	6.82	4.30	6.34	3.89	5.08	2.79	3.98	1.96	3.11	1.32	2.11	0.77	1.60	0.57	1.28	0.46	1.09	0.29
	7	5.14	2.86	5.12	2.81	4.82	2.54	4.00	1.92	4.04	1.92	3.20	1.39	1.79	0.63	1.34	0.48	1.10	0.30	1.01	0.11
2.00	3	4.37	2.59	4.38	2.64	4.29	2.55	4.01	2.34	3.58	1.97	3.21	1.68	2.51	1.18	2.02	0.88	1.66	0.67	1.40	0.54
	5	2.98	1.45	2.97	1.43	2.92	1.41	2.77	1.29	2.58	1.17	2.35	1.04	1.90	0.77	1.57	0.62	1.32	0.49	1.15	0.35
	7	2.38	1.03	2.36	1.02	2.33	0.98	2.24	0.93	2.26	0.93	2.12	0.86	1.62	0.62	1.33	0.49	1.15	0.36	1.04	0.20
2.50	3	2.84	1.53	2.82	1.55	2.80	1.52	2.71	1.46	2.57	1.35	2.46	1.27	2.14	1.05	1.86	0.87	1.52	0.68	1.42	0.58
	5	2.01	0.89	2.00	0.90	1.98	0.88	1.95	0.87	1.88	0.83	1.80	0.78	1.63	0.68	1.44	0.58	1.28	0.47	1.15	0.36
	7	1.64	0.67	1.64	0.67	1.64	0.66	1.60	0.65	1.60	0.65	1.56	0.63	1.39	0.54	1.24	0.44	1.13	0.34	1.05	0.22
3.00	3	2.16	1.11	2.16	1.12	2.12	1.09	2.09	1.07	2.07	1.06	2.00	1.01	1.84	0.89	1.67	0.78	1.61	0.70	1.38	0.58
	5	1.58	0.67	1.59	0.69	1.57	0.67	1.57	0.68	1.55	0.66	1.49	0.62	1.40	0.57	1.32	0.51	1.22	0.44	1.14	0.35
	7	1.32	0.51	1.33	0.51	1.32	0.50	1.32	0.50	1.31	0.50	1.30	0.49	1.21	0.42	1.14	0.36	1.09	0.29	1.04	0.21

**Table 3:** The ARL and SDRL results of proposed CC using P distribution under LLF, with $\lambda$ = 0.10.

$\beta$	$n$	$\alpha$ = 0.00		$\alpha$ = 0.10		$\alpha$ = 0.25		$\alpha$ = 0.50		$\alpha$ = 0.75		$\alpha$ = 1.00		$\alpha$ = 1.50		$\alpha$ = 2.00		$\alpha$ = 2.50		$\alpha$ = 3.00	
		ARL	SDRL	ARL	SDRL	ARL	SDRL	ARL	SDRL	ARL	SDRL	ARL	SDRL	ARL	SDRL	ARL	SDRL	ARL	SDRL	ARL	SDRL
0.25	3	5.14	0.90	5.16	0.90	5.16	0.91	5.15	0.91	5.15	0.88	4.82	0.57	3.23	0.42	2.70	0.45	2.00	0.00	2.00	0.00
	5	3.38	0.51	3.38	0.51	3.37	0.50	3.37	0.50	3.37	0.50	3.36	0.49	2.87	0.33	2.00	0.00	2.00	0.00	1.59	0.49
	7	2.84	0.37	2.83	0.38	2.83	0.37	2.83	0.38	2.84	0.37	2.82	0.37	2.01	0.10	2.00	0.00	1.73	0.44	1.00	0.00
0.50	3	10.95	3.81	10.89	3.80	10.89	3.72	10.03	2.85	7.26	1.42	5.25	0.88	3.36	0.50	2.60	0.49	2.01	0.11	1.99	0.02
	5	6.29	1.53	6.27	1.52	6.26	1.52	6.13	1.33	5.14	0.83	3.95	0.57	2.72	0.45	2.00	0.07	1.99	0.08	1.55	0.49
	7	4.80	0.99	4.80	0.98	4.81	0.99	4.77	0.94	4.19	0.62	3.29	0.46	2.12	0.33	1.99	0.03	1.64	0.47	1.00	0.07
0.75	3	47.14	35.27	46.04	34.52	34.27	22.43	13.61	5.49	7.67	2.24	5.34	1.27	3.41	0.64	2.58	0.51	2.07	0.26	1.98	0.14
	5	20.12	11.01	20.20	11.12	17.38	8.17	9.13	2.86	5.52	1.34	4.00	0.82	2.67	0.51	2.04	0.20	1.94	0.23	1.52	0.49
	7	13.81	6.23	13.82	6.27	12.47	4.99	7.26	2.01	4.56	0.99	3.36	0.62	2.23	0.42	1.97	0.15	1.57	0.21	1.06	0.23
0.9	3	246.78	234.91	171.84	156.35	47.67	36.29	13.83	6.60	7.70	2.68	5.38	1.52	3.44	0.76	2.58	0.55	2.10	0.31	1.96	0.19
	5	130.63	120.74	95.02	83.05	28.40	18.41	9.45	3.69	5.60	1.64	4.05	0.98	2.68	0.57	2.06	0.27	1.90	0.29	1.51	0.49
	7	84.62	70.81	63.14	50.95	20.92	11.99	7.54	2.58	4.60	1.19	3.38	0.74	2.26	0.44	1.95	0.22	1.56	0.49	1.09	0.29
1.00	3	369.75	360.99	180.41	170.66	45.16	35.47	13.84	7.18	7.73	2.94	5.38	1.66	3.45	0.82	2.60	0.58	2.13	0.36	1.94	0.26
	5	369.41	349.08	133.91	123.49	28.27	19.47	9.42	3.96	5.59	1.78	4.05	1.07	2.67	0.59	2.08	0.31	1.88	0.32	1.51	0.49
	7	370.76	362.83	105.50	93.12	21.11	12.96	7.58	2.84	4.63	1.31	3.41	0.83	2.28	0.47	1.93	0.26	1.56	0.49	1.11	0.32
1.25	3	33.54	25.64	30.91	22.92	22.16	15.00	12.02	6.54	7.53	3.30	5.40	2.03	3.50	1.04	2.62	0.66	2.17	0.45	1.92	0.36
	5	19.61	12.51	18.52	11.75	14.57	8.36	8.60	3.98	5.56	2.04	4.08	1.29	2.72	0.70	2.12	0.43	1.83	0.39	1.51	0.50
	7	14.45	8.43	14.06	7.95	11.38	5.84	6.95	2.83	4.63	1.54	3.44	1.00	2.33	0.53	1.90	0.36	1.54	0.49	1.17	0.37
1.50	3	12.46	7.46	12.28	7.18	11.34	6.44	9.00	4.61	6.70	3.07	5.25	2.14	3.50	1.18	2.67	0.78	2.19	0.54	1.91	0.45
	5	7.94	3.75	7.82	3.74	7.45	3.37	6.22	2.57	4.96	1.89	3.96	1.35	2.73	0.79	2.14	0.51	1.81	0.45	1.52	0.50
	7	6.07	2.61	6.05	2.52	5.81	2.31	5.09	1.87	4.11	1.39	3.32	1.04	2.34	0.60	1.88	0.44	1.55	0.50	1.22	0.41
2.00	3	5.67	2.68	5.68	2.71	5.51	2.58	5.15	2.36	4.71	2.07	4.21	1.76	3.32	1.27	2.64	0.92	2.20	0.69	1.90	0.58
	5	3.81	1.42	3.80	1.44	3.75	1.43	3.60	1.34	3.41	1.23	3.12	1.08	2.70	0.83	2.11	0.63	1.78	0.55	1.52	0.52
	7	3.09	1.04	3.07	1.03	3.06	1.04	2.98	0.97	2.83	0.90	2.63	0.81	2.20	0.64	1.83	0.53	1.53	0.51	1.27	0.44
2.50	3	3.81	1.72	3.77	1.67	3.77	1.66	3.66	1.61	3.50	1.48	3.31	1.38	2.87	1.14	2.47	0.94	2.14	0.79	1.87	0.67
	5	2.66	0.95	2.66	0.96	2.65	0.95	2.61	0.94	2.53	0.91	2.45	0.85	2.56	0.82	1.95	0.65	1.71	0.59	1.50	0.54
	7	2.19	0.71	2.22	0.72	2.20	0.71	2.16	0.69	2.14	0.68	2.06	0.64	1.89	0.58	1.68	0.56	1.48	0.52	1.28	0.45
3.00	3	2.93	1.30	2.93	1.29	2.89	1.25	2.87	1.22	2.81	1.23	2.69	1.14	2.49	1.04	2.22	0.89	2.02	0.80	1.81	0.72
	5	2.11	0.77	2.11	0.77	2.09	0.75	2.07	0.74	2.05	0.73	2.01	0.72	1.88	0.68	1.73	0.63	1.59	0.58	1.43	0.52
	7	1.76	0.60	1.77	0.61	1.74	0.60	1.74	0.61	1.73	0.60	1.70	0.59	1.61	0.56	1.49	0.54	1.37	0.49	1.24	0.43

**Table 4:** The run length profiles for Bayesian Max EWMA CC using P distribution under LLF, with $\lambda$ = 0.25.

$\beta$	$n$	$\alpha$ = 0.00		$\alpha$ = 0.10		$\alpha$ = 0.25		$\alpha$ = 0.50		$\alpha$ = 0.75		$\alpha$ = 1.00		$\alpha$ = 1.50		$\alpha$ = 2.00		$\alpha$ = 2.50		$\alpha$ = 3.00	
		ARL	SDRL	ARL	SDRL	ARL	SDRL	ARL	SDRL	ARL	SDRL	ARL	SDRL	ARL	SDRL	ARL	SDRL	ARL	SDRL	ARL	SDRL
0.25	3	4.51	1.20	4.51	1.22	4.48	1.19	4.52	1.21	4.48	1.17	4.00	0.71	2.70	0.45	2.00	0.00	1.94	0.23	1.02	0.16
	5	2.66	0.53	2.64	0.53	2.64	0.53	2.64	0.53	2.64	0.53	2.62	0.49	1.99	0.02	1.83	0.37	1.00	0.01	1.00	0.00
	7	2.06	0.27	2.06	0.25	2.06	0.25	2.06	0.26	2.06	0.26	2.03	0.20	1.99	0.06	1.00	0.09	1.00	0.00	1.00	0.00
0.50	3	14.58	9.36	14.78	9.51	14.69	9.46	13.51	8.12	7.51	2.80	4.56	1.15	2.63	0.53	2.01	0.14	1.77	0.41	1.17	0.37
	5	5.84	2.29	5.81	2.23	5.86	2.27	5.64	2.02	4.34	1.07	3.13	0.60	2.02	0.18	1.68	0.46	1.03	0.18	1.00	0.01
	7	4.07	1.21	4.04	1.20	4.07	1.22	4.02	1.12	3.36	0.69	2.54	0.52	1.93	0.24	1.12	0.32	1.00	0.00	1.00	0.00
0.75	3	115.74	110.06	117.17	109.94	91.83	85.91	21.30	15.67	7.83	3.80	4.65	1.64	2.69	0.68	2.03	0.35	1.70	0.45	1.27	0.44
	5	37.50	31.80	36.84	30.93	30.75	24.55	10.27	5.64	4.85	1.74	3.22	0.90	2.07	0.36	1.62	0.48	1.12	0.32	1.00	0.05
	7	19.80	14.68	19.70	14.57	17.09	11.85	7.03	3.12	3.76	1.15	2.62	0.66	1.84	0.38	1.22	0.41	1.00	0.00	1.00	0.00
0.9	3	275.23	270.77	236.46	227.03	102.7	97.16	18.34	13.53	7.62	4.08	4.68	1.94	2.72	0.79	2.03	0.44	1.67	0.48	1.29	0.45
	5	253.44	244.45	197.15	187.86	52.61	47.39	10.18	6.17	4.92	2.07	3.27	1.07	2.08	0.47	1.60	0.49	1.16	0.36	1.01	0.12
	7	173.83	169.45	130.75	125.32	33.79	28.28	7.35	3.75	3.82	1.35	2.66	0.77	1.82	0.43	1.26	0.44	1.02	0.14	1.00	0.02
1.00	3	370.61	367.61	249.47	244.13	74.47	70.65	16.71	12.60	7.47	4.17	4.63	2.02	2.74	0.85	2.05	0.52	1.66	0.49	1.31	0.46
	5	369.33	363.61	197.64	196.45	43.43	39.11	9.87	6.24	4.87	2.15	3.27	1.15	2.09	0.54	1.60	0.50	1.18	0.39	1.02	0.15
	7	370.60	365.94	164.13	161.02	30.40	25.18	7.18	3.96	3.83	1.48	2.69	0.84	1.81	0.47	1.27	0.44	1.02	0.16	1.00	0.03
1.25	3	36.48	33.09	33.79	30.23	23.98	20.18	11.74	8.49	6.78	4.04	4.56	2.24	2.77	1.02	2.07	0.65	1.65	0.54	1.35	0.48
	5	21.49	17.67	20.26	16.75	14.63	11.26	7.72	4.86	4.69	2.36	3.27	1.34	2.12	0.68	1.59	0.53	1.23	0.42	1.05	0.23
	7	15.16	11.61	14.51	10.85	11.20	7.97	5.96	3.28	3.78	1.66	2.70	0.98	1.79	0.56	1.32	0.47	1.06	0.24	1.00	0.05
1.50	3	11.55	8.73	11.34	8.56	10.05	7.41	7.54	5.02	5.58	3.28	4.22	2.23	2.77	1.18	2.06	0.76	1.66	0.59	1.37	0.50
	5	6.87	4.38	6.71	4.15	6.30	3.79	5.05	2.82	3.89	1.89	3.09	1.33	2.09	0.78	1.60	0.57	1.27	0.45	1.09	0.28
	7	5.19	2.85	5.10	2.83	4.81	2.55	4.06	1.99	3.23	1.40	2.56	0.99	1.78	0.62	1.34	0.49	1.09	0.29	1.01	0.12
2.00	3	4.46	2.66	4.39	2.62	4.30	2.55	4.04	2.29	3.59	1.99	3.18	1.69	2.50	1.18	2.01	0.87	1.65	0.68	1.41	0.55
	5	2.94	1.43	2.95	1.45	3.90	2.01	2.77	1.29	2.57	1.16	2.34	1.02	1.90	0.78	1.57	0.62	1.31	0.49	1.14	0.35
	7	2.37	1.02	2.37	1.00	2.35	1.01	2.25	0.94	2.12	0.86	1.96	0.77	1.62	0.62	1.34	0.50	1.14	0.35	1.04	0.20
2.50	3	2.82	1.55	2.81	1.54	2.80	1.52	2.72	1.44	2.59	1.35	2.44	1.25	2.13	1.04	1.85	0.84	1.61	0.70	1.41	0.58
	5	1.99	0.89	2.00	0.90	2.93	1.40	1.94	0.86	1.89	0.83	1.82	0.78	1.62	0.68	1.44	0.58	1.27	0.47	1.16	0.37
	7	1.65	0.68	1.66	0.68	1.64	0.68	1.62	0.66	1.58	0.64	1.51	0.61	1.38	0.53	1.25	0.44	1.13	0.34	1.05	0.23
3.00	3	2.16	1.13	2.17	1.15	2.13	1.09	2.11	1.10	2.06	1.06	2.00	1.00	1.83	0.89	1.67	0.78	1.51	0.68	1.37	0.58
	5	1.59	0.69	1.59	0.69	1.56	0.67	1.56	0.67	1.53	0.65	1.49	0.64	1.40	0.57	1.30	0.50	1.21	0.43	1.13	0.35
	7	1.32	0.51	1.33	0.52	1.33	0.51	1.31	0.50	1.30	0.49	1.27	0.47	1.21	0.42	1.15	0.37	1.09	0.29	1.04	0.21

Likewise, Tables 3 and 4 display the ARL results of the offered Bayesian Max-EWMA CC using the LLF with a fixed smoothing constant value of 0.25 and a sample size of 5. We considered various shifts in both the process i.e., a = 0.00, 0.25, 0.50, 0.75, 1.50, 3.00, along with the corresponding shift in the process variance i.e., *b* = 1 and obtained corresponding ARL values of 370.61, 44.74, 9.85, 4.96, 2.10, and 1.02. These results illustrate that as the process shifts increase, the ARL values decrease rapidly, indicating the accurate performance of the suggested Max-EWMA CC in detecting shifts in both the process mean and variance. Furthermore, it is important to note that the efficiency of the proposed CC for jointly monitoring the process mean and variance depends on the sample size. In Table 5, we have compared the suggested Bayesian Max-EWMA CC with the existing Bayesian EWMA CC using different values of smoothing constants i.e., $\lambda$ = 0.10, 0.15 and 0.25 and with sample size *n* = 5. The ARL outcomes clearly shows that the proposed Bayesian Max-EWMA CC is more significantly identify signals indicating an out-of-control state more effectively than the existing Bayesian EWMA CC. From all the Tables 1, 2, 3, 4 and 5, it is evident that as the sample size increases, the ARL outcomes decrease, indicating the greater efficiency of the suggested CC in detecting deviations from the expected process parameters. The key findings of the study are given as:

- The efficiency of the suggested Max-EWMA CC for simultaneously monitoring the process mean and variance, and for detecting minor to moderate shifts, is evident from the run length profiles presented in all four tables associated with the suggested CC.
- Based on the simulation results, it is observed that the performance of the suggested Bayesian CC for joint monitoring improves as the smoothing constant value decreases.
- In the current study, one of the most crucial factors under consideration is the variability in sample size. The results obtained from our analysis provide a clear and compelling insight. It is evident that as the sample size increases, the effectiveness and performance of the suggested Bayesian Max-EWMA CC experience a substantial and notable improvement.

**Table 5:** The ARL and SDRL results for Bayesian EWMA and Bayesian Max-EWMA CC using P distribution under LLF, with $\lambda$ = 0.10.

Shift	$\lambda$ = 0.10,* n* = 5				$\lambda$ = 0.15,* n* = 5				$\lambda$ = 0.25,* n* = 5			
	Bayesian EWMA (Riaz et al.^13^)		Proposed Bayesian Max-EWMA		Bayesian EWMA (Riaz et al.^13^)		Proposed Bayesian Max-EWMA		Bayesian EWMA (Riaz et al.^13^)		Proposed Bayesian Max-EWMA	
	ARL	SDRL	ARL	SDRL	ARL	SDRL	ARL	SDRL	ARL	SDRL	ARL	SDRL
0.0	370.12	367.89	369.22	366.19	370.94	365.44	368.07	363.96	369.49	364.82	370.61	369.43
0.10	251.51	244.31	138.44	127.94	269.37	261.69	157.62	149.44	287.66	281.31	202.33	201.85
0.20	125.58	114.98	42.70	32.40	143.05	134.99	50.95	44.01	178.20	175.14	71.14	66.83
0.25	89.67	80.09	28.63	19.50	104.95	96.58	32.09	24.91	135.62	133.07	44.74	39.57
0.30	66.57	57.92	20.74	12.61	78.89	71.10	22.57	16.12	104.70	100.95	29.61	24.95
0.40	41.68	32.78	13.11	6.61	48.17	41.84	13.28	7.91	63.11	58.20	15.51	11.54
0.50	28.35	20.12	9.47	4.02	31.95	25.54	9.29	4.65	41.21	36.61	9.87	6.24
0.60	20.98	13.49	7.40	2.73	22.95	16.87	7.02	2.96	28.45	24.57	7.03	3.80
0.70	16.26	9.59	6.13	2.04	17.10	11.50	5.71	2.20	20.61	16.37	5.48	2.62
0.80	13.41	7.14	5.21	1.57	13.69	8.55	4.75	1.63	15.71	11.75	4.47	1.88
0.90	11.42	5.69	4.57	1.29	11.29	6.54	4.12	1.31	12.51	8.86	3.75	1.44
1.00	9.78	4.50	4.04	1.08	9.57	5.11	3.63	1.07	10.22	6.77	3.28	1.17
1.50	5.79	2.03	2.69	0.60	5.38	2.15	2.39	0.55	5.15	2.51	2.1	0.53
2.00	4.16	1.20	2.09	0.32	3.78	1.22	1.93	0.34	3.46	1.33	1.60	0.50
2.50	3.31	0.84	1.88	0.33	2.97	0.84	1.56	0.49	2.66	0.86	1.19	0.39
3.00	2.76	0.66	1.53	0.49	2.47	0.62	1.16	0.37	2.19	0.61	1.02	0.15
4.00	2.12	0.38	1.01	0.12	1.96	0.38	1.00	0.02	1.66	0.50	1.00	0.00
5.00	1.89	0.32	1.00	0.00	1.62	0.48	1.00	0.00	1.27	0.44	1.00	0.00

## Real life data application

This article presents the practical implementation of the proposed Bayesian Max-EWMA CC. The data used for this demonstration is drawn from Montgomery^21^ and pertains to the hard-bake process in semiconductor manufacturing. The dataset consists of 45 samples, each containing 5 wafers, resulting in a total of 225 data points. These measurements, in microns, represent the flow width, and the time interval between each sample is consistently set at 1 h. Of these samples, the initial 30, comprising 150 observations, are considered indicative of a controlled process and are labeled as the phase-I dataset. Conversely, the remaining 15 samples, totaling 75 observations, are designated as representative of an out-of-control process and are referred to as the phase-II and the complete dataset is available in the appendix A. Both charts are employed to monitor variations in the process mean, and the computed results are showcased in Table 6.

**Table 6:** The values and out of control status Bayesian EWMA CC and proposed Bayesian Max-EWMA under SELF, with $\lambda$ = 0.10.

Sample #	Existing Bayesian EWMA	UCL	Out-of-control status	Proposed Bayesian Max-EWMA	UCL	Out-of-control status
1	1.4999	1.5389	0	0.9660	4.247	0
2	1.4997	1.5389	0	1.6490	4.247	0
3	1.4988	1.5389	0	2.1420	4.247	0
4	1.5054	1.5389	0	2.7406	4.247	0
5	1.5039	1.5389	0	2.9623	4.247	0
6	1.5099	1.5389	0	3.2743	4.247	0
7	1.5079	1.5389	0	3.4687	4.247	0
8	1.5058	1.5389	0	3.4954	4.247	0
9	1.5077	1.5389	0	3.4932	4.247	0
10	1.5075	1.5389	0	3.5513	4.247	0
11	1.5068	1.5389	0	3.5463	4.247	0
12	1.5078	1.5389	0	3.6801	4.247	0
13	1.5090	1.5389	0	3.7683	4.247	0
14	1.5079	1.5389	0	3.7473	4.247	0
15	1.5032	1.5389	0	3.7101	4.247	0
16	1.5033	1.5389	0	3.9640	4.247	0
17	1.5032	1.5389	0	3.8458	4.247	0
18	1.5078	1.5389	0	3.7488	4.247	0
19	1.5025	1.5389	0	3.6464	4.247	0
20	1.5044	1.5389	0	3.7663	4.247	0
21	1.5039	1.5389	0	3.6734	4.247	0
22	1.5077	1.5389	0	3.7575	4.247	0
23	1.5056	1.5389	0	3.7666	4.247	0
24	1.5066	1.5389	0	3.6643	4.247	0
25	1.5082	1.5389	0	3.6609	4.247	0
26	1.5098	1.5389	0	3.6587	4.247	0
27	1.5065	1.5389	0	3.6872	4.247	0
28	1.5109	1.5389	0	3.9081	4.247	0
29	1.5081	1.5389	0	3.8826	4.247	0
30	1.5083	1.5389	0	3.7623	4.247	0
31	1.5075	1.5389	0	3.7277	4.247	0
32	1.5107	1.5389	0	3.7382	4.247	0
33	1.5124	1.5389	0	3.6431	4.247	0
34	1.5171	1.5389	0	3.5679	4.247	0
35	1.5220	1.5389	0	3.5307	4.247	0
36	1.5178	1.5389	0	3.5486	4.247	0
37	1.5211	1.5389	0	3.5551	4.247	0
38	1.5216	1.5389	0	4.0309	4.247	0
39	1.5237	1.5389	0	**4.4998**	**4.247**	**1**
40	1.5216	1.5389	0	**4.9497**	**4.247**	**1**
41	1.5211	1.5389	0	**5.3826**	**4.247**	**1**
42	1.5203	1.5389	0	**5.7985**	**4.247**	**1**
43	1.5235	1.5389	0	**6.1951**	**4.247**	**1**
44	1.5200	1.5389	0	**6.5784**	**4.247**	**1**
45	1.5193	1.5389	0	**6.5884**	**4.247**	**1**

Significant values are in bold

**Figure 1:**
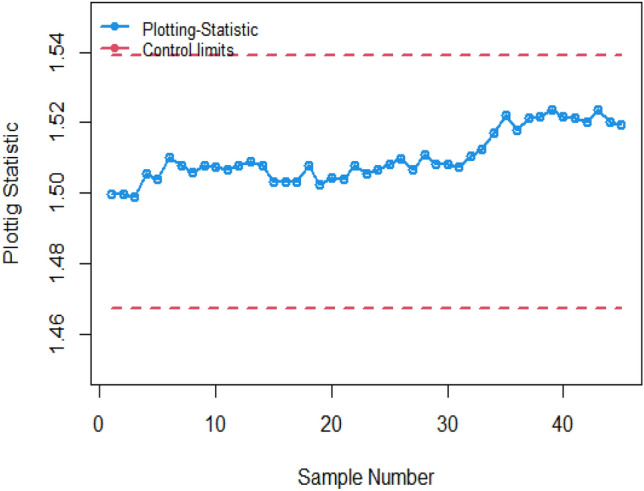
Using SELF, the Bayesian EWMA CC with $\lambda = 0.10$.

**Figure 2:**
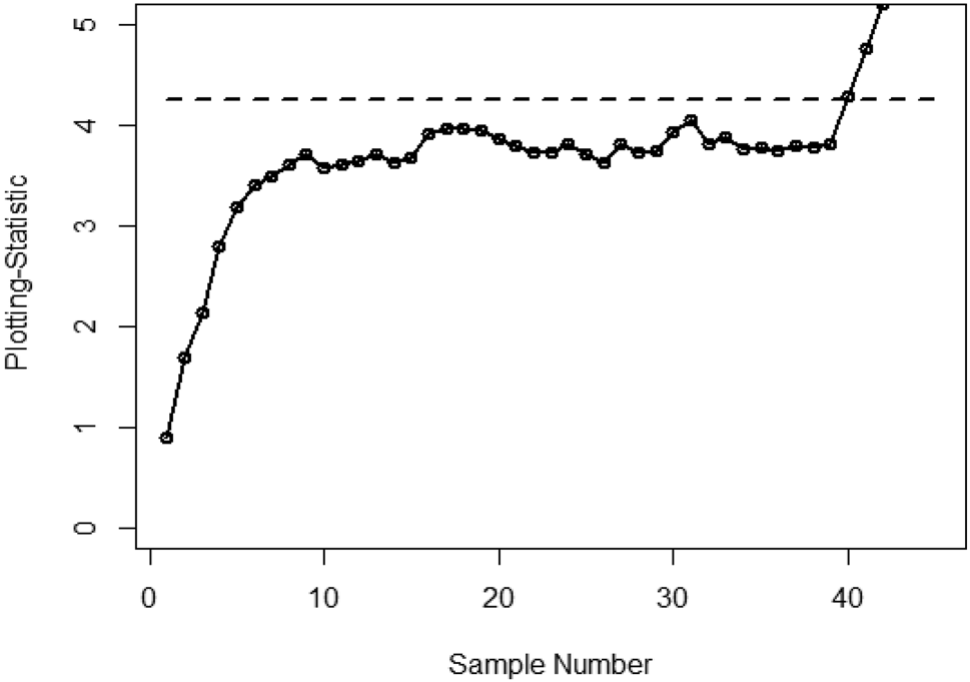
Under SELF, the Bayesian Max-EWMA control chart for jointly monitoring with $\lambda = 0.10$.

**Figure 3:**
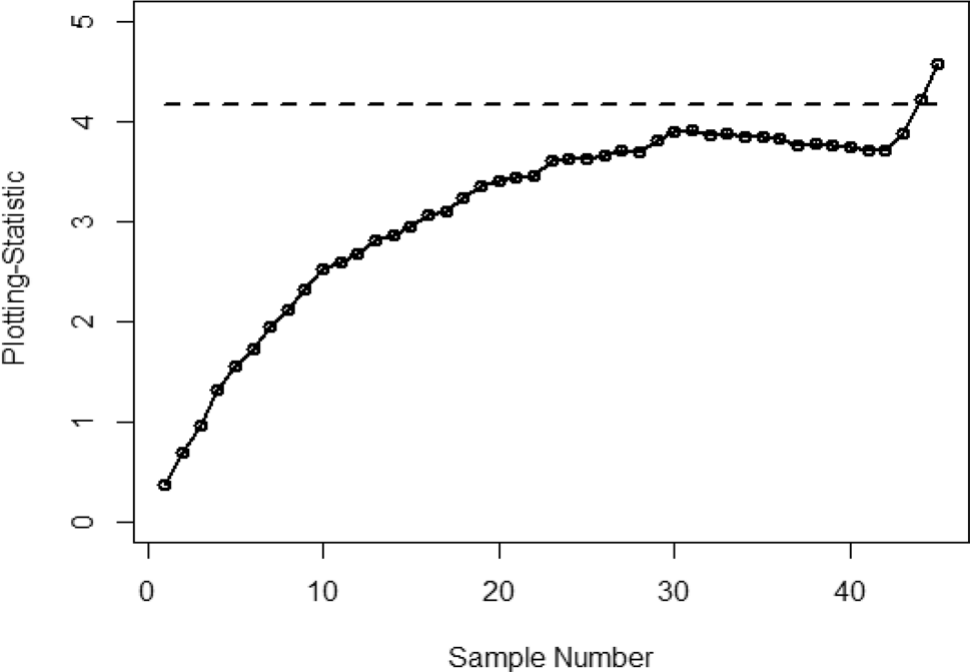
Using SELF, the Bayesian Max-EWMA CC for jointly monitoring with $\lambda = 0.25$.

**Figure 4:**
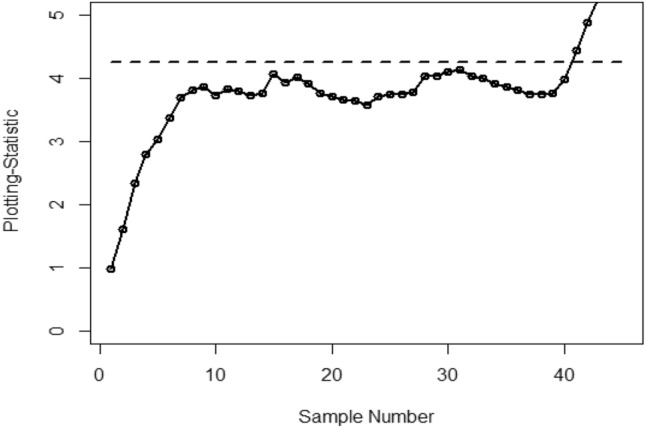
Bayesian Max-EWMA CC for jointly monitoring using LLF with $\lambda = 0.10$.

**Figure 5:**
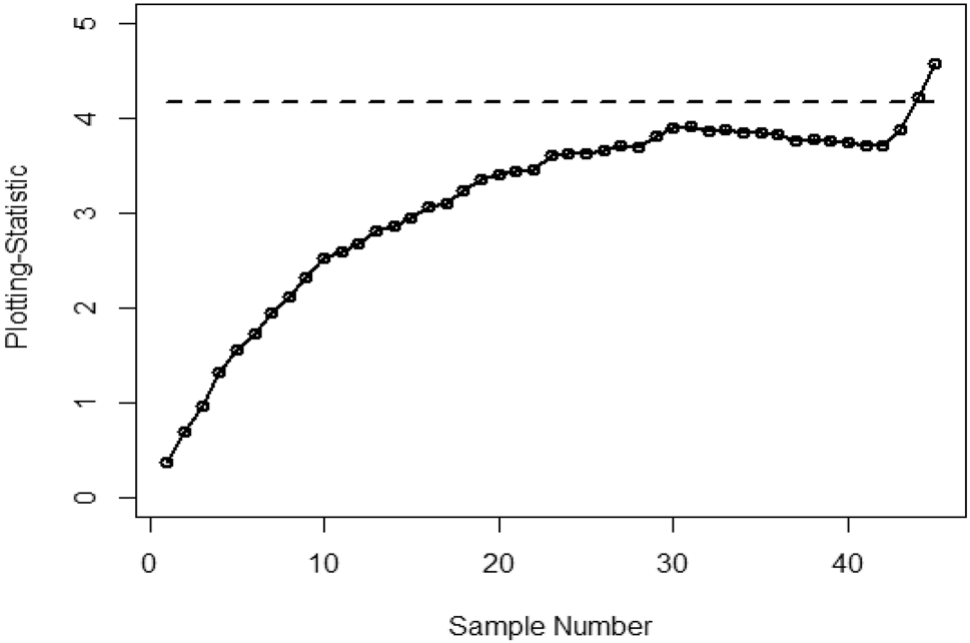
Using LLF, Bayesian Max-EWMA CC for jointly monitoring with $\lambda = 0.25$.

Figure 1 shows the Bayesian EWMA CC under SELF, in which all the points are within the control. Figures 2 and 3 provide a visual representation of the implementation of the recommended Bayesian Max-EWMA CC, designed to jointly monitor both the process mean and dispersion using the SELF approach. Upon closer examination of these charts, it becomes apparent that the process exhibits signals indicating it is out of control in the 39th and 43rd samples, for the smoothing constant values 0.10 and 0.25 respectively. Similarly, Figs. 4 and 5 depict the performance of the proposed CC using the LLF approach. These figures clearly show that the process displays out-of-control signals in the 40th and 42nd samples within the same context. This observation not only underscores the effectiveness of the Bayesian Max-EWMA CC but also indicates that the performance of the suggested CC deteriorates as the smoothing constant value increases.

## Conclusion

This study introduces an innovative Bayesian Max-EWMA CC designed for concurrent monitoring of both process mean and variance. It utilizes informative prior distributions and incorporates two distinct LFs within the context of P distributions. The results, presented in Tables 1, 2, 3, and 4, evaluate the performance of the proposed CC using metrics such as ARL and SDRL. ARL plots (Figs. 1, 2, 3, 4) provide compelling evidence of the superior performance of the Bayesian CC. To further assess the CC under varying LFs, a practical example is applied to the semiconductor manufacturing hard bake process. Notably, the proposed Bayesian Max-EWMA CC, for both P distributions, excels in detecting out-of-control signals. Importantly, the principles of this study can be extended to other memory-type CCs.

Moreover, this approach is not confined to normal distributions; it can be tailored for data conforming binomial or Poisson distributions, albeit requiring adjustments to the likelihood function. Expanding this innovative technique to non-normal distributions and various CC types can yield a more comprehensive understanding of underlying data. This, in turn, facilitates early detection of potential quality issues, enables swift corrective actions, and reduces the risk of costly errors and defects. In practical applications, such as healthcare, this approach aids in promptly identifying anomalies in patient data, allowing for timely interventions. Within finance, it has the ability to reveal fraudulent activities and potential errors in financial transactions. In manufacturing, broadening the scope of this approach to encompass to non-normal distributions and various CC types helps in detecting variations in the production process, elevating product quality and reducing waste.

### Supplementary Information

Supplementary Information.

## Data Availability

The corresponding author holds the datasets utilized or analyzed in the ongoing study and can grant access to interested parties upon a reasonable request. This process ensures that those seeking access to the data for further examination or validation purposes can communicate with the corresponding author to obtain the necessary information.
