# Supplementary material for: A novel Bayesian Max-EWMA control chart for jointly monitoring the process mean and variance: an application to hard bake process
Source: Sci Rep. 2023 Dec 1;13:21224. doi: 10.1038/s41598-023-48532-4 (PMC10692141; doi:10.1038/s41598-023-48532-4)
Supplement: Supplementary file 1 — Supplementary Information. [file 41598_2023_48532_MOESM1_ESM.docx]

**Dataset**

1.5119,1.4951,1.4817,1.4712,1.4882,1.4492,1.5805,1.5343,1.5076,1.5134,1.5242,1.5284,1.3947,1.5261,1.4083,1.5344,1.4874,1.4573,1.5777,1.5060,1.4691,1.5390,1.5592,1.5688,1.5264,1.4998,1.5142,1.5332,1.4152,1.5097,1.4724,1.5292,1.5317,1.5793,1.4279,1.4894,1.4980,1.6198,1.6630,1.6490,1.6786,1.6322,1.7040,1.6391,1.7770
